# Adaptation of the professionalism mini-evaluation exercise instrument into Turkish: a validity and reliability study

**DOI:** 10.1186/s12909-023-04675-6

**Published:** 2023-09-26

**Authors:** Ali İhsan Taşçı, Esra Akdeniz, Mehmet Ali Gülpınar, Yavuz Onur Danacıoğlu, Emine Ergül Sarı, Levent Yaşar, Faruk Karandere, Sina Ferahman

**Affiliations:** 1 School of Medicine, Department of Medical Education, Marmara University, Istanbul, Turkey; 2 Department of Urology, Bakirkoy Dr. Sadi Konuk Training and Research Hospital, Istanbul, Turkey; 3 Department of Pediatric Diseases, Bakirkoy Dr. Sadi Konuk Training and Research Hospital, Istanbul, Turkey; 4 Department of Gynecology and Obstetrics, Bakirkoy Dr. Sadi Konuk Training and Research Hospital, Istanbul, Turkey; 5 Department of Internal Medicine, Bakirkoy Dr. Sadi Konuk Training and Research Hospital, Istanbul, Turkey; 6 Department of General Surgery, Bakirkoy Dr. Sadi Konuk Training and Research Hospital, Istanbul, Turkey

**Keywords:** Medical professionalism, Residency, Education, Educational measurement

## Abstract

**Background:**

There is an ongoing search for standardized scales appropriate for each culture to evaluate professionalism, which is one of the basic competencies of a physician. The Professionalism Mini-evaluation Exercise (P-MEX) instrument was originally developed in Canada to meet this need. In this study, it was aimed to adapt the P-MEX to Turkish and to evaluate the validity and reliability of the Turkish version.

**Methods:**

A total of 58 residents at Bakirkoy Dr. Sadi Konuk Training and Research Hospital were assessed with the Turkish version of P-MEX by 24 raters consisting of faculty members, attending physicians, peer residents, and nurses during patient room visits, outpatient clinic and group practices. For construct validity, the confirmatory factor analysis was performed. For reliability, Cronbach’s alpha scores were calculated. Generalizibility and decision studies were undertaken to predict the reliability of the validated tool under different conditions. After the administration of P-MEX was completed, the participants were asked to provide feedback on the acceptability, feasibility, and educational impact of the instrument.

**Results:**

A total of 696 forms were obtained from the administration of P-MEX. The content validity of P-MEX was found to be appropriate by the faculty members. In the confirmatory factor analysis of the original structure of the 24-item Turkish scale, the goodness-of-fit parameters were calculated as follows: CFI = 0.675, TLI = 0.604, and RMSEA = 0.089. In the second stage, the factors on which the items loaded were changed without removing any item, and the model was modified. For the modified model, the CFI, TLI, and RMSEA values were calculated as 0.857, 0.834, and 0.057, respectively. The decision study on the results obtained from the use of P-MEX in a Turkish population revealed the necessity to perform this evaluation 18 times to correctly evaluate professionalism with this instrument. Cronbach’s alpha score was 0.844. All the faculty members provided positive feedback on the acceptability, feasibility, and educational impact of the adapted P-MEX.

**Conclusion:**

The findings of this study showed that the Turkish version of P-MEX had sufficient validity and reliability in assessing professionalism among residents. Similarly, the acceptability and feasibility of the instrument were found to be high, and it had a positive impact on education.

**Trial registration:**

2020/249, Bakirkoy Dr. Sadi Konuk Training and Research Hospital.

## Introduction

Professionalism is one of the main competencies of physicians. Professional incompetence has many negative consequences, including reduced quality of healthcare, increased dissatisfaction, conflicts, and violence, as well as decreasing the value and prestige of the medical profession. Therefore, the evaluation of professionalism is of great importance in medical education [1, 2].

There is no consensus on the definition and framework of professionalism, with various definitions having been made according to different perspectives [3–9]. Therefore, learning and assessment methods are also diverse. Epstein and Hundert define professionalism as “the continuous and reasonable reflection of communication skills, professional knowledge, technical skills, clinical reasoning, emotions, and values into daily practice for the benefit of the individuals and community being served”. This not only covers the concept of professionalism and its components as in other definitions but also emphasizes social expectations and culture [10, 11].

In the report of the International Working Group on the Assessment of Professionalism published in 2011, the main themes and recommendations were determined, and the need to endorse a multidimensional perspective was emphasized to evaluate professionalism at individual, interpersonal, and social-institutional levels [12]. In 2019, this group evaluated the studies on the subject and stated that the main uncertainty and field of study concerned the definition and evaluation of professionalism [13].

It is not possible for any measurement and evaluation tool/method to assess professionalism with all its dimensions. Therefore, it is recommended to evaluate the personal, interpersonal, and social/institutional dimensions of professionalism using different tools and approaches to increase consolidation [14]. Although various tools have been developed to assess professionalism, the lack of valid and reliable standard assessment tools remains a major challenge [12].

The characteristics of professionalism may vary according to culture, and therefore the acceptance and feasibility of tools and methods developed to measure and assess professionalism in one culture may pose problems in another culture. Likewise, the translations of the definition of professionalism into other languages may not reflect their original meanings. For these reasons, if any scale is to be used in another language and culture, validity and reliability studies should first be undertaken [12].

As in other measurements and evaluations, the main quality indicators of the assessment of professionalism are reliability, validity, acceptability, feasibility, and the educational impact of methods and tools [15–17]. Studies on consensus-based standards for the selection of health measurement instruments (COSMIN) guide the evaluation of the quality of measurement tools [17–19]. However, it has been stated that there is not yet a systematic assessment method for the assessment of the quality of measurement characteristics of instruments measuring medical professionalism based on a universally accepted standard framework, which reduces confidence in professionalism measurement tools and their results [20].

The Professionalism Mini-evaluation Exercise (P-MEX) is an instrument developed in the mini-clinical evaluation exercise (mini-CEX) format to assess professionalism. A study group of 92 faculty members at McGill University (Canada) identified 142 observable behaviors reflecting professionalism, and then created a four-point scale with 24 of these behaviors. Rating in this instrument is based on the following four levels: 4, above expectations; 3, met expectations; 2, below expectations; and 1, unacceptable. In addition, the option ‘not observed’/ ‘not applicable’ was added to the instrument [21].

In the original study, P-MEX was used first by 38 faculty members having observed a student in a simulated environment, and then by four faculty members from the internal diseases department having observed a patient-student encounter. According to the feedback received, the form was revised and used to assess third- and fourth-year medical students in internal medicine, general surgery, pediatric, psychiatry, and obstetric clinics. A total of 211 forms were collected as a result of the assessment of 74 students [21].

In the item analysis of P-MEX, four items for which the ‘not applicable’ option was selected by 40% of the raters were considered to be not suitable for the instrument. In addition, the items frequently marked as ‘below expectations’ were interpreted to be more sensitive in showing professionalism violations, and some items were deemed unnecessary due to their close correlation with other items. The explanatory factor analysis showed that the scale consisted of four factors: doctor–patient relationship skills, reflective skills, time management, and interprofessional relationship skills. According to the generalizability analysis and decision study, 10–12 P-MEX forms were found to be necessary to accurately assess professionalism. At the end of semi-structured interviews, the authors stated that P-MEX assessments stimulated self-reflection, increased the importance of professionalism, learning, and awareness of behaviors appropriate for professionalism. P-MEX has been prepared for use in any environment where student-patient encounters take place and are suitable for observation by the evaluator [21].

To date, the validation of the P-MEX instrument has been undertaken in two cultures: Japanese and Iranian. The scale was tested with residents in the Japan study in 2009, residents and fellows in the study in 2011, and residents in the Iran study in 2019 [22–24]. Another study showed a correlation between the P-MEX evaluations performed before residency and at the end of the first residency year were correlated [25]. Also, an adaptation study was carried out with the use of P-MEX in a simulated environment [26].

This study aimed to adapt the P-MEX instrument into Turkish and evaluate the validity and reliability of the Turkish version. The research questions determined for this purpose are as follows:

1. Is the Turkish version of P-MEX appropriate for the Turkish culture?
2. How many P-MEX forms are necessary to accurately assess professionalism?
3. What are the acceptability, feasibility, and educational impact of P-MEX?

## Material and method

This observational study was conducted at the University of Health Sciences, Bakirkoy Dr. Sadi Konuk Training and Research Hospital in 2021–2022. Approval was obtained from the ethics committee of the hospital (2020/249).

P-MEX has been translated into Turkish separately by five experts in the field of language and education. These translations were evaluated by the panel of authors (AIT, EA, MAG, YOD) and were made into a single translation by consensus. The first version of Turkish P-MEX was translated back into English by native English linguists. Differences in meaning between the back-translation and the original scale were evaluated and the Turkish P-MEX was prepared by making the necessary corrections.

The Turkish p-MEX scale used in this study, like the original scale, consists of 24 items in four categories: doctor–patient relationship skills, reflective skills, time management, and interprofessional skills. Likewise, the likert scale of the original scale was used where 4, above expectations; 3, met expectations; 2, below expectations; and 1, unacceptable. In addition, the option 'not observed'/'not applicable' was added to the instrument.

### Participants

The study included a total of 60 residents from five clinics, 10 from the general surgery clinic, 10 from the obstetrics and gynecology clinic, 10 from the urology clinic, 20 from the internal medicine clinic, and 10 from the pediatric health and diseases clinic. In order to strengthen inclusivity and diversity in terms of clinics, five departments have been identified from surgery and internal medicine clinics. These departments were determined based on the health care differences of clinics, the patient population served, the size of the clinic and the number of residents. Afterwards, residents who accepted to work on a voluntary basis from these selected departments were included in the study.

The power analysis is based on detecting a misspecified model where effect measure is taken as root mean squared error of approximation (RMSEA). The required sample size (N) for alpha 0.05, power 0.80, RMSEA effect size 0.02 and degrees of freedom 236 is 629 [27].

As evaluators, a total of 24 individuals (four from each clinic) were selected from faculty members, attending physicians, peer residents, and nurses, who had worked with the residents for at least three months. Among the evaluators, residency program managers, professors, associate professors were defined as the faculty, physicians and specialists working in the clinic as attending physicians, residents working in the same clinic as peer residents, and nurses working in the same clinic as nurses.

### Training and preliminary study

Training sessions of at least one hour each were held with the residents and evaluators under the leadership of the researcher at the clinics where the study was to be conducted. In these sessions, the purpose of the study, characteristics of the Turkish version of P-MEX, and the assessment process were explained. In addition, an informative brochure of the research and an informed consent form were distributed to the participants. During these sessions, the participants were also informed that all the evaluations to be made by the evaluators would be kept confidential, and they were free to withdraw from the study at any stage. After the training sessions, a resident- patient encounter lasting for at least 20 min was watched by the evaluator group, and a preliminary application was undertaken by asking the evaluators to assess the residents using the Turkish version of P-MEX. At the end of the training and pre-study, the questions of the trainers were answered and feedback was given about the pre-application.

### Data collection

Evaluations were made in three settings: patient rooms where resident-patient encounters took place (patient room visits), outpatient clinic rooms (outpatient practices), and meeting rooms (group practices). In all three settings, each resident was evaluated with the Turkish version of P-MEX by a total of four evaluators, including a faculty member, an attending physician, a peer resident, and a nurse three times in a minimum of 20-min a resident-patient encounters over a period of at least one month. All evaluators (faculty members, relevant doctors, peer assistants, and nurses) responsible for the evaluation of residents observed and scored the resident-patient encounter separately or made independent observation and scoring by having more than one evaluator in an application.

After all the P-MEX forms were completed, the content validity of the scale was investigated by asking the 6 faculty members (Residency program administrators, professors, associate professors, who were evaluators in the study) and a faculty member from the medical education department with five-point Likert scale, whether they agreed that the content of the P-MEX instrument was appropriate for assessing the professional competencies of physicians and covered areas related to professionalism, as well as presenting them with two open-ended questions: “In your opinion, what items should be added to the P-MEX instrument to better assess the professional competencies of physicians?” and “In your opinion, which items should be removed from the P-MEX instrument due to not being appropriate for observation or measuring similar characteristics to other items?”. In addition, the faculty members and residents were asked to complete feedback forms with five-point Likert scale to elicit their views on the acceptability, feasibility, and educational impact of P-MEX.

### Data analysis

Descriptive statistics were used to present data on the demographic characteristics of the participants, evaluation settings, and item analyses.

Construct validity for all the P-MEX items was evaluated with the confirmatory factor analysis (CFA) through structural equation modeling (SEM). The model’s goodness-of-fit status was investigated with the comparative fit index (CFI), Tucker-Lewis index (TLI), and the root mean square error of approximation (RMSEA). Jamovi 2.3.21 and R 4.3.0 (‘lavaan’ package) were used for the analysis of construct validity.

The reliability of the results obtained from the Turkish culture was further examined with a generalizability analysis and a decision study. The dependability coefficient was determined with the crossed design, in which the residents were the object of measurement applications. The decision study was conducted to determine how many times the P-MEX instrument should be administered to accurately assess professionalism. In this calculation, a dependability coefficient (phi) of 0.80 was accepted to provide appropriate reproducibility [28]. The generalizability analysis and decision study were undertaken using R software (‘gtheory’ package).

Cronbach’s alpha scores were calculated to evaluate the internal consistency of the instrument. Internal consistency analyses were performed using the ‘psychometry’ package of R software. Power analysis for SEM was conducted using ‘semPower’ package.

Lastly, we examined the responses of the faculty members to the concerning content of P-MEX,

and the responses of the faculty members and residents to the items in the feedback forms concerning the acceptability, feasibility, and educational impact of the instrument.

## Results

In this study, 58 of 60 residents were evaluated using the Turkish version of P-MEX three times in three different clinical settings (patient room visits, outpatient clinic practices, and group practices) by a total of 24 evaluators consisting of faculty members, attending physicians, peer residents, and nurses (Table 1). The evaluation of the remaining two residents could not be completed since they changed their institution during the study period. A total of 696 P-MEX forms were completed. Twenty-six of the residents were female (44.82%), and 32 were male (55.17%).

**Table 1:** Distribution of the residents and evaluators by clinic

	Clinic	Resident	Evaluator
Surgical diseases	Gynecology and obstetrics	9	4
	General surgery	10	4
	Urology	10	4
Internal diseases	Pediatric health and diseases	10	4
	Internal medicine (A and B)	19	8
**Total**		**58**	**24**

### Item analyses

The mean P-MEX score of the 696 forms was calculated as 3.2 (SD: 0.2). Table 2 presents the mean ± standard deviation score of each item.

**Table 2:** P-MEX item analyses^a^

Factor	Item	Item definitions	Mean (SD)
I- Doctor-patient relationship skills	P1	Listened actively to patient	3.4 (0.5)
	P2	Showed interest in patient as a person	3.3 (0.5)
	P3	Showed respect for patient	3.3 (0.5)
	P4	Recognized and met patient needs	3.2 (0.5)
	P5	Accepted inconvenience to meet patient needs	3.1 (0.6)
	P6	Ensured continuity of patient care	3.2 (0.5)
	P7	Advocated on behalf of a patient and/or family member	3.1 (0.5)
	P12	Maintained appropriate boundaries with patients/colleagues	3.4 (0.5)
II- Reflective skills	P8	Demonstrated awareness of limitations	3.3 (0.6)
	P9	Admitted errors/omissions	3.1 (0.6)
	P10	Solicited feedback	2.8 (0.6)
	P11	Accepted feedback	3.2 (0.5)
	P13	Maintained composure in a difficult situation	3.2 (0.5)
III- Time management	P15	Was on time	3.3 (0.6)
	P16	Completed tasks in a reliable fashion	3.4 (0.5)
	P18	Was available to patients or colleagues	3.4 (0.5)
IV- Interprofessional skills	P12	Maintained appropriate boundaries with patients/colleagues	3.4 (0.5)
	P14	Maintained appropriate appearance	3.4 (0.6)
	P17	Addressed own gaps in knowledge and skills	3.1 (0.6)
	P19	Demonstrated respect for colleagues	3.6 (0.5)
	P20	Avoided derogatory language	3.6 (0.5)
	P21	Assisted a colleague as needed	3.4 (0.5)
	P22	Maintained patient confidentiality	3.3 (0.5)
	P23	Used health resources appropriately	3.3 (0.5)
	P24	Respected rules and procedures of the system	3.3 (0.5)
**Mean P-MEX score**			**3.2 (0.2)**

^a^Factors and items ordered according to the original scale [20]

*P-MEX* Professionalism Mini-evaluation Exercise

**Fig. 1:**
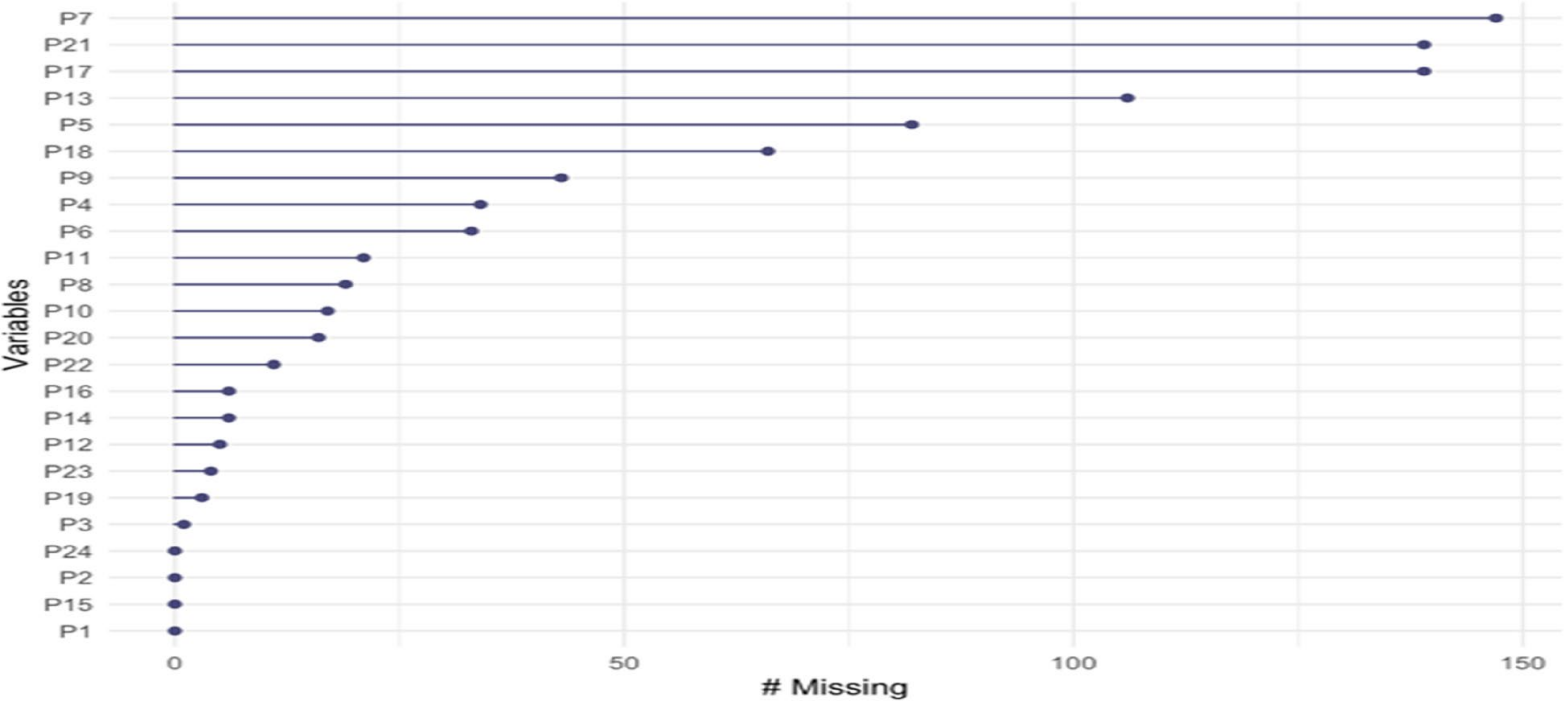
Number and rates of items marked as ‘not observed’/ ‘not applicable’

**Fig. 2:**
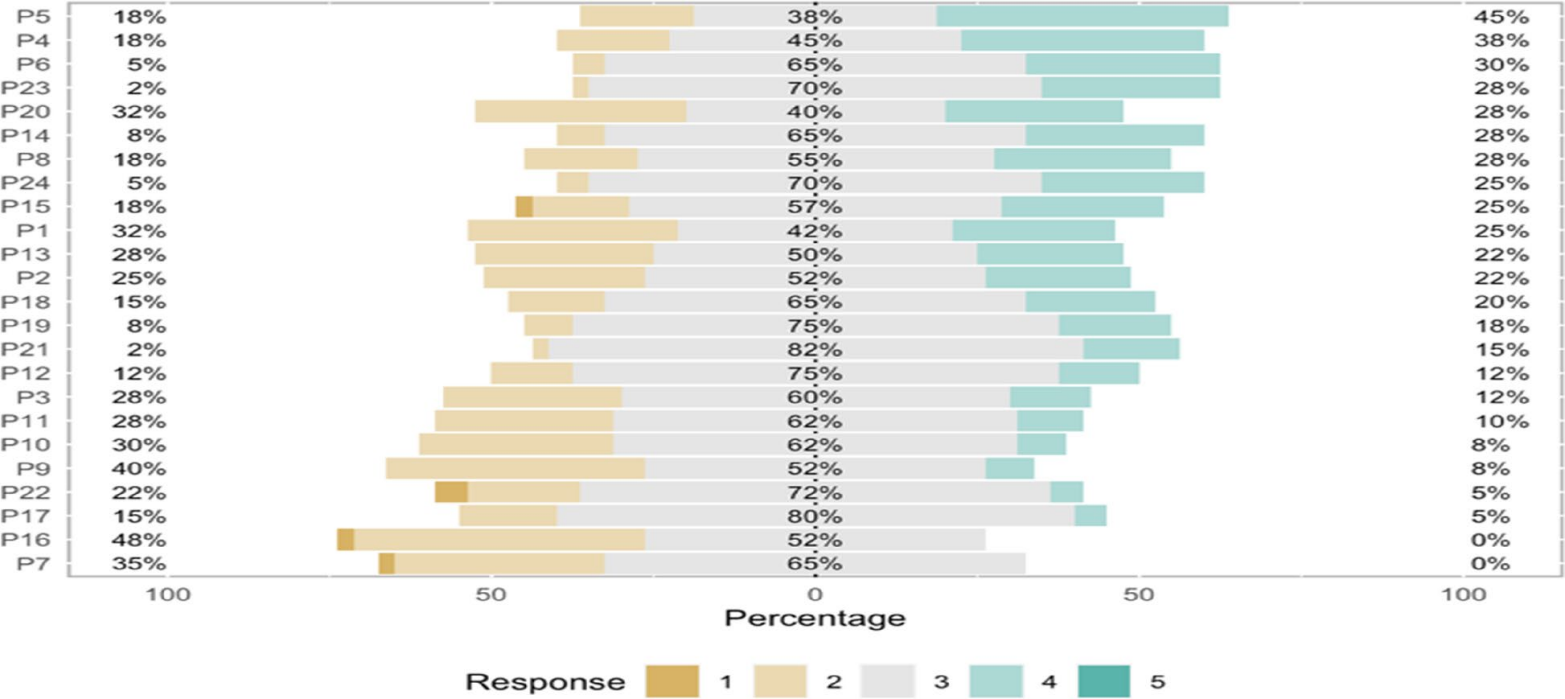
Likert plot of P-MEX items (positive to negative ordered from top to bottom)

Among the P-MEX items, the ‘not observed’/’not applicable’ option was most marked for P7 (“advocated on behalf of a patient and/or family member”) (21.12%), followed by P17 (“addressed own gaps in knowledge and skills”) and P21 (“assisted a colleague as needed”) (19.97% for both). The most positive items were determined as P5 and P4, and the most negative items were P7 and P16 (Figs. 1 and 2).

When the surgical and internal diseases clinics were evaluated separately, the mean P-MEX score of the former was statistically significantly higher than that of the latter (*p* = 0.023).

When each clinic was evaluated separately, the mean P-MEX score was calculated as 3.1 ± 0.2 for the gynecology and obstetrics clinic, 3.4 ± 0.2 for the general surgery clinic, 3.5 ± 0.1 for the urology clinic, 3.2 ± 0.2 for the pediatric health and diseases clinic, and 3.2 ± 0.2 for the internal medicine clinic. There was a statistically significant difference between the P-MEX scores of the clinics (*P* < 0.001). Pairwise comparisons were undertaken to determine which clinics had significant differences in the P-MEX scores. Accordingly, only the mean scores of the pediatric health and diseases clinic and the internal medicine clinic were similar. The order of the clinics from the highest to the lowest P-MEX scores was as follows: urology, general surgery, pediatric health and diseases, internal medicine, and gynecology and obstetrics.

Considering the evaluator groups, the mean P-MEX score was calculated as 3.3 ± 0.2 for the faculty members, 3.2 ± 0.2 for the attending physicians, 3.3 ± 0.3 for the peer residents, and 3.3 ± 0.2 for the nurses, indicating statistically significant differences (P < 0.001). Pairwise comparisons were performed to identify the groups with significant differences. While the P-MEX scores of the faculty members and nurses did not significantly differ, the attending physicians and peer residents had significantly different P-MEX scores. From the highest to the lowest P-MEX scores, the order of the evaluator groups was as follows: peer residents, faculty members, nurses, and attending physicians (Table 3).

**Table 3:** Comparison of the P-MEX scores by evaluator group

	**Peer residents (*n* = 174)**	**Faculty members (*n* = 174)**	**Nurses (*n* = 174)**	**Attending physicians (*n* = 174)**	**Total (*n* = 696)**	***p***
Mean (SD)	3.3 (0.3)^ab^	3.3 (0.2)^a^	3.3 (0.2)^a^	3.2 (0.2)^b^	3.2 (0.2)	< 0.001^1^
Min–max	2.8—4.0	2.8—3.7	2.8—3.7	2.7—3.6	2.7—4.0	

^a,b^Different letters denote statistically significant differences in pairwise comparisons (*p* < 0.05)

^1^Analysis of variance test p value

*P-MEX* Professionalism Mini-evaluation Exercise, *mean* arithmetic mean, *SD* Standard deviation, *min* minimum, *max* maximum

When the P-MEX scores were analyzed according to the evaluation setting, the mean score was determined as 3.2 ± 0.3 for group practices, 3.3 ± 0.3 for patient room visits, and 3.2 ± 0.2 for outpatient clinic practices. The mean P-MEX scores did not significantly differ according to the evaluation setting (Table 4, *P* = 0.196).

**Table 4:** Comparison of the P-MEX scores by evaluation setting

	**Group practices (*n* = 232)**	**Outpatient practices (*n* = 232)**	**Patient room visits (*n* = 232)**	**Avarage (*n* = 696)**	***p***
Mean (SD)	3.2 (0.3)	3.2 (0.2)	3.3 (0.3)	3.2 (0.2)	0.196^1^
Min–max	2.8—3.9	2.8—3.8	2.7—4.0	2.7—4.0	

*P-MEX* Professionalism Mini-evaluation Exercise, *mean* arithmetic mean, *SD* Standard deviation, *min* minimum, *max* maximum

^1^t-test *p* value

When analyzed according to gender, the mean P-MEX score of the male residents was statistically significantly higher than that of the female residents (3.3 ± 0.3 and 3.2 ± 0.2, respectively; *P* = 0.017.

### Validity

**Fig. 3:**
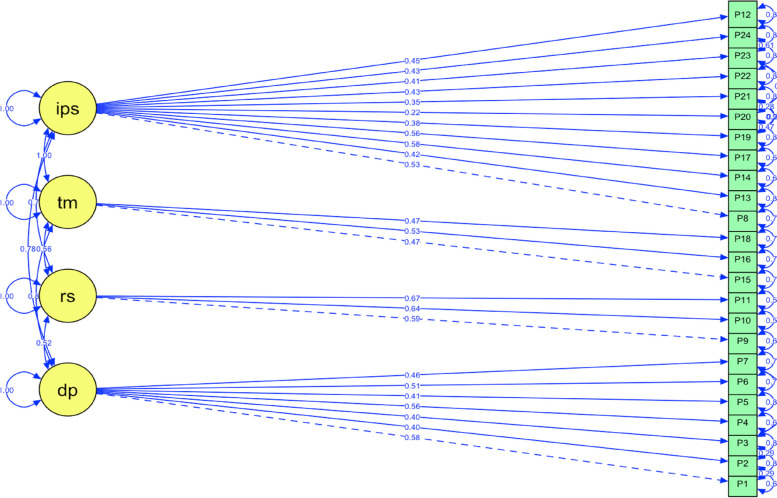
Diagram of the confirmatory factor analysis through the structural equation modeling of the modified P-MEX instrument. dp: doctor-patient relationship skills, rs: reflective skills, tm: time management, ips: interprofessional skills. One-way arrows represent causal relationships between the variables, while double-headed arrows represent correlations between two variables. The numbers presented next to the arrows indicate standard path coefficients. The goodness-of-fit values of the model were determined as follows: CFI = 0.857, TLI = 0.834, and RMSEA = 0.057

In the confirmatory factor analysis performed to evaluate the construct validity of the 24-item Turkish version of P-MEX in relation to the original scale structure, CFI was calculated as 0.675, TLI as 0.604, and RMSEA as 0.089. No item was removed from the modified model. Only the factors on which the items loaded were changed, with the covariances of the items within the same factor being added to the model (P23 ~  ~ P24, P19 ~  ~ P20, P2 ~  ~ P3, P1 ~  ~ P2, P3 ~  ~ P7, P20 ~  ~ P21, P19 ~  ~ P21, and P20 ~  ~ P23). The CFI, TLI, and RMSEA values of the modified model were determined as 0.857, 0.834, and 0.057 respectively (Table 5) (Fig. 3).

**Table 5:** CFA goodness-of-fit indicators of the models

	**CFI**	**TLI**	**RMSEA**	**RMSEA 90% CI**	
				**Lower bound**	**Upper bound**
Model: Original	0.648	0.604	0.089	0.082	0.095
Model: Modified	0.857	0.834	0.057	0.049	0.065

*CFI* Comparative fit index, *TLI* Tucker-Lewis index, *RMSEA* Root mean square error of approximation, *CI* Confidence interval

“The content of the P-MEX instrument is appropriate for assessing the professional competencies of physicians” and “The items of the P-MEX instrument cover all areas related to professionalism to assess the professional competencies of physicians”. The faculty members had a high level of agreement with these statements (with five-point Likert scale 4.83 ± 0.4 and 4.66 ± 0.5, respectively). When asked which other items should be added to the P-MEX instrument to assess the professional competencies of physicians, the faculty members referred to items related to medical interview skills, speaking effectively and clearly, informing the patient and obtaining consent, and awareness of responsibility, in order of frequency. When asked which items should be removed from the P-MEX instrument due to not being appropriate for observation or measuring similar characteristics to other items, the faculty members mostly responded as P7 (“advocated on behalf of a patient and/or family member”) and P11 (“accepted feedback”).

### Reliability

The generalizability analysis and decision study were performed with R software (gtheory package), and the results are presented in Table 6 in comparison with the original P-MEX study and the Japanese adaptation study. It was observed that a dependability coefficient of 0.80 and above was achieved by 12 P-MEX forms with the original scale, 16 forms with the Japanese version, and 18 forms with the Turkish version.

**Table 6:** Results of the Turkish P-MEX decision study and comparison with other studies

Number of evaluations	Dependability coefficient		
	Turkish P-MEX	Japanese P-MEX [21]	Original P-MEX [20]
1	0.37	0.22	0.28
2	0.52	0.36	0.44
4	0.65	0.53	0.61
6	0.70	0.62	0.70
8	0.73	0.69	0.76
10	0.75	0.73	0.79
12	0.77	0.77	0.82
14	0.78	0.79	0.84
16	0.79	0.81	
18	0.80		

*P-MEX* Professionalism Mini-evaluation Exercise

### Internal consistency

For all the items in the P-MEX instrument, the Cronbach alpha was calculated as 0.844. Concerning the four dimensions presented in the original scale, the Cronbach alpha values were determined as 0.641 for doctor-patient relationship skills, 0.62 for reflective skills, 0.401 for time management, and 0.684 for interprofessional skills (Table 7).

**Table 7:** P-MEX factors and internal consistency values of the original and modified P-MEX

Factors	Items (original)	Cronbach alpha (original)	Items (modified)	Cronbach alpha (modified)
Overall scale	P1-P24	0.844	P1-P24	0.844
Doctor-patient relationship skills	P1, P2, P3, P4, P5, P6, P7, P12	0.641	P1, P2, P3, P4, P5, P6, P7	0.646
Reflective skills	P8, P9, P10, P11, P13	0.620	P9, P10, P11	0.614
Time management	P15, P16, P18	0.401	P15, P16, P18	0.401
Interprofessional skills	P12, P14, P17, P19, P20, P21, P22, P23, P24	0.684	P8, P12, P13, P14, P17, P19, P20, P21, P22, P23, P24	0.734

P12, which was included in both the ‘doctor-patient relationship skills’ and ‘interprofessional skills’ dimensions in the original scale, was only in the ‘interprofessional skills’ dimension in the modified version. In addition, P8 and P13, which were in the ‘reflective skills’ dimension in the original scale, was included in the ‘interprofessional skills’ dimension in the modified version

*P-MEX* Professionalism Mini-evaluation Exercise

### Acceptability, feasibility, and educational impact

All the faculty members provided positive feedback concerning the acceptability and educational impact of the P-MEX instrument. However, considering feasibility, none of the faculty members agreed with the statements, “The time required to assess professionalism with the P-MEX instrument is not too long” and “It is easy to use the P-MEX instrument in outpatient clinics” (Table 8).

**Table 8:** Feedback of faculty members on the acceptability, feasibility, and educational impact of the P-MEX instrument

	Statements	Five-point Likert-type score^a^, Mean (SD)
Acceptability	The P-MEX instrument is a valuable method to assess residents’ professional competence and improve their development	4.66 (0.5)
	The P-MEX instrument should be used to assess residents’ professional competencies and ensure their development	4.5 (0.5)
Feasibility	The number of questions in the P-MEX instrument is not too high	2.33 (0.5)
	The items on the P-MEX instrument can be easily understood	3.83 (0.4)
	It is easy to assess residents with the P-MEX instrument	3.83 (0.4)
	The time required to assess professionalism with the P-MEX instrument is not too long	1.83 (0.4)
	I had no difficulty in allocating the necessary time to use the P-MEX instrument	3.83 (0.9)
	It is easy to use the P-MEX instrument during patient room visits	4.16 (0.4)
	It is easy to use the P-MEX instrument in outpatient clinics	1.83 (0.4)
	It is easy to use the P-MEX instrument in group practices	4.5 (0.5)
	The time I spend on using the P-MEX instrument does not affect my performance score in providing health care	2.83 (0.7)
	P-MEX is a useful method to assess residents’ professional competence	3.83 (0.4)
Educational impact	Assessment of professionalism with the P-MEX instrument increases the importance of professionalism	4.16 (0.4)
	P-MEX can help identify the professional deficiencies of residents and provides an opportunity to overcome them	4.66 (0.5)
	With the P-MEX instrument, it is easier to provide feedback after the assessment of professionalism	4.83 (0.4)
	Assessment of professionalism with P-MEX positively affects education	4.83 (0.4)

^a^5- strongly agree, 4- agree, 3- neither agree nor disagree, 2- disagree, 1- strongly disagree

*P-MEX* Professionalism Mini-evaluation Exercise, *SD* Standard deviation

The residents also expressed positive views concerning the acceptability, feasibility, and educational impact of the P-MEX instrument. Of the residents, only 17% each did not agree with the statements, “The time required to assess professionalism with the P-MEX instrument is not too long” and “P-MEX assessment increases my motivation to act professionally” (Table 9).

**Table 9:** Resident feedback on the acceptability, feasibility, and educational impact of the P-MEX instrument

		Five-point Likert-type score^a^, Mean (SD)
Acceptability	The P-MEX instrument is a valuable method to assess professional competence	3.98 (0.7)
	The P-MEX instrument should be used to assess professional competence	3.72 (0.9)
Feasibility	The items on the P-MEX instrument can be easily understood	4.23 (0.6)
	The time required to assess professionalism with the p-MEX instrument is not too long	3.58 (1)
	The P-MEX instrument is a useful method to assess professionalism	3.69 (0.8)
Educational impact	I have increased awareness of the importance of professionalism after P-MEX assessment	3.81(0.7)
	I have become more aware of my positive and negative characteristics after P-MEX assessment	3.74 (0.7)
	P-MEX assessment increases my motivation to act professionally	3.34 (1)

^a^5- strongly agree, 4- agree, 3- neither agree nor disagree, 2- disagree, 1- strongly disagree

*P-MEX* Professionalism Mini-evaluation Exercise, *SD* Standard deviation

## Discussion

There is a need for valid, reliable, acceptable, and feasible scales with positive educational effects to assess professionalism in medicine. However, difficulties remain in the development of standard scales that can be used in different cultures and educational environments [12]. In our validation study of the Turkish adaptation of the P-MEX instrument, which was originally developed by Cruess et al. in Canada, we obtained adequate validity and reliability findings. We also determined that the faculty members and residents had generally positive views concerning the acceptability, feasibility, and educational impact of the P-MEX instrument [21].

Before commencing the research, we determined the clinics and residents to participate in the study, P-MEX evaluators, and when and how many times P-MEX assessments would be undertaken, and all the study stages were in compliance with these predefined protocols. In this regard, our research differs from previous studies [21–24].

In the original P-MEX study, the authors found that among the P-MEX items, P5 (“accepted inconvenience to meet patient needs”), P7 (“advocated on behalf of a patient and/or family member”), P9 (“admitted errors/omissions”), and P21 (“assisted a colleague as needed”) might not be suitable for the instrument since they were marked as ‘not observed’/ ‘not applicable’ at a rate of more than 40% [21]. In the current study, in relation to P7, P17, and P21, the option ‘not observed’/ ‘not applicable’ was selected at a rate of approximately 20%, which is lower compared to the original study.

Items marked as ‘below expectations’/ ‘unacceptable’ in the P-MEX instrument are important in terms of showing deficiencies in students’ professional competence. In the original P-MEX study, four items, namely “demonstrated awareness of limitations” (P8), “solicited feedback” (P10), “was on time” (P15), and “addressed own gaps in knowledge and skills” (P17) were marked as ‘below expectations’/ ‘unacceptable’ at a higher rate than the remaining items [21]. In the Japanese P-MEX study, it was stated that the scores of the items “ensured continuity of patient care” (P6), “solicited feedback” (P10), “was on time” (P15), and “addressed own gaps in knowledge and skills knowledge and skills” (P17) had lower scores compared to the other items [22]. In the current study, the lowest scoring items were determined as P7 (“admitted errors/omissions”) and P16 (“completed tasks in a reliable fashion”).

One of the differences of our study is that we also investigated the differences in the mean P-MEX scores according to the clinics where the residents were doing their residency, evaluator groups, evaluation settings, and gender of the participants. Content validity can be determined by asking subject experts whether the items in a scale cover all the features to be measured and whether there are items that need to be added or removed [18, 29, 30]. The expert group needs to have detailed knowledge of the characteristics that the scale intends to measure. However, the possibility of increased bias should be taken into account when content validity is undertaken by the same team that also designed the elements of the measurement tool [30].

In our study, according to expert opinion, it was necessary to add further items to the scale to address medical interview skills, speaking effectively and clearly, informing patients and obtaining their consent, and awareness of responsibility, while the items “advocated on behalf of a patient and/or family member” (P7) and “accepted feedback” (P11) were suggested to be removed. In the P-MEX validation study conducted in Japan in 2009, the authors stated that the following four items should be added to the scale to achieve content validity: “respect for different opinions”, “asking for expert opinion when necessary”, “good medical practice”, and “obtaining informed consent” [22]. In a more recent study, Fong et al. assessed the content validity of original P-MEX and suggested that four items, “solicited feedback” (P10), “accepted inconvenience to meet patient needs” (P5), “advocated on behalf of a patient and/or family member” (P7), and “maintained appropriate appearance” (P14), were not appropriate and could be removed from the instrument, while there was a need to add new items on collegiality and communication with empathy. These discrepancies between studies in relation to the items that need to be added or removed to achieve content validity may be associated with cultural differences [31, 32]. Experts in the residence program in Singapore reached consensus that 19 of the p-MEX items are suitable for assessing professionalism. However, they could not reach a consensus on the inclusion or exclusion of four items (solicited feedback, advocated on behalf of a patient, extended his/herself to meet patient needs, used health resources appropriately) from the scale [33]. In Asian culture, unlike western countries, respect for patients, accountability and reliability emerge as the main elements of professionalism [34] These discrepancies between studies in relation to the items that need to be added or removed to achieve content validity may be associated with cultural differences [35].

The best professionalism assessment can be made by direct observation of the student-patient encounter by the evaluator [36]. Collecting data from multiple observers in different situations increases the validity of the results [37]. Evaluations can be made by educators, physicians, peers, nurses and patients [38]. The bias of evaluations made by peer residents is a matter of debate [39, 40]. In our study, the scores given by the peer evaluators were higher than other evaluators. Scales were developed to assess medical professionalism by patients [41, 42] However, it is stated that they should be used with caution due to the limitations of patient feedback [36]. In our study, the fact that P-MEX applications were made with the direct observation of residents of different status evaluators, excluding patients, is in line with the basic principles mentioned above.

Construct validity is examined by CFA through structural equation modeling. In this analysis, a CFI value of > 0.90 and RMSEA value of < 0.05 indicate an appropriate fit and a value of < 0.10 for both indicates an acceptable fit [43, 44]. In CFA we performed on the original structure of the 24-item Turkish version of P-MEX, we found these values to be low. However, the construct validity criteria of the model modified without item removal was of acceptable level.

Item 12 in the original P-MEX instrument (“maintained appropriate boundaries with patients/colleagues”) was equally classified into two dimensions: doctor-patient relationship skills and interprofessional skills. In our study, this item was determined to be unidimensional since it had a higher factor loading for interprofessional skills. Similar findings were reported by Tsugawa et al. [22, 23]. In addition, we observed that P8 and P13, which were included in the reflective skills factor in the original scale, loaded on the interprofessional skills factor in the Turkish version.

The reliability of a test includes its consistency, reproducibility, and generalizability [45]. According to the Mini-CEX study, a dependability coefficient of 0.80 indicates good reproducibility [28]. In the original P-MEX study, it was stated that 10–12 P-MEX forms were required to provide a dependability coefficient of 0.80 [21]. The first P-MEX validation study performed by Tsugawa et al. in 2009 revealed that 16 forms were required to achieve reliability [22]. In their second study conducted in 2011, Tsugawa et al. reported that the number of P-MEX forms required was 6–8 for evaluator clinicians, 4–6 for nurses, and 26 for peer residents and junior doctors [23]. Our result was higher, indicating that 18 P-MEX assessments were required for the appropriate measurement of professional competence.

Internal consistency is a measure of the extent to which the items of a scale are related (homogeneous) and measure the same concept. Internal consistency is considered appropriate when Cronbach’s alpha is between 0.70 and 0.95 [17]. In our study, the Cronbach alpha value for all the items in the P-MEX instrument was calculated as 0.844. The lower Cronbach alpha values ​​for the dimensions as presented in the original scale may be related to low number of items in these dimensions.

In this study, the questions and items prepared to elicit the views of the faculty members and residents concerning the acceptability, feasibility, and educational impact of P-MEX were based on theoretical foundations in these fields [15, 16]. While the participants expressed positive views on the acceptability and educational impact of the adapted scale, when asked about feasibility, they considered that it took too long to apply the instrument and it was difficult to use it in outpatient settings. Similarly, in the original P-MEX study, the major limitation of the instrument was reported to be the time-consuming nature of observing students, recording results, and providing feedback [21]. In a study from Iran, it was emphasized that P-MEX assessment and feedback were negatively affected during peak hours in emergency clinics [24]. In another P-MEX study conducted with residents in Singapore, 113 (34%) of the 333 participants stated that the instrument was too long to administer [31]. Kaur et al., assessing the professional characteristics of dental students in India with P-MEX, reported that 71.43% of the students and 75% of the faculty members considered the instrument to be feasible, while 23.81% of the students stated that it was too long. Concerning acceptability, 61.9% of the students and 75% of the faculty members felt comfortable with the use of P-MEX, while 33% of the students felt anxiety during assessment [46]. In relation to the educational impact of P-MEX, the original study showed that this instrument increased the reflection and awareness of professionalism and facilitated the recognition of unprofessional behaviors [21]. In the P-MEX study conducted in Japan, 83% of the students reported that the assessment result was consistent with their self-evaluation, 70% considered that the P-MEX assessment motivated them to act professionally, 70% agreed that the scale items were reasonable and appropriate to assess professionalism, and 61% thought that this self-assessment experience helped see themselves more objectively [22]. In the current study, while the participants had positive views concerning the acceptability and educational impact of P-MEX, from the feasibility perspective, they had some negative opinions, addressing the importance of not only the structure of the instrument but also problems in its application in terms of clinical burden, clinical service environment, and healthcare system conditions. Based on this feedback, in addition to revisions to be made to the structure of the instrument, there is also a need to address the optimization of clinical service load of residents to provide qualified education.

One of the major limitations of this study is that people who are under observation and know that they are being assessed tend not to exhibit their real behaviors and modify them, as is the case in all observational evaluations. In addition, the study was conducted with residents in a single center, which may affect the generalizability of the results; therefore, there is a need for multicenter studies.

## Conclusion

The findings obtained from this study showed that the Turkish version of the P-MEX instrument is valid, reliable, acceptable, and feasible and has positive effects on education in the Turkish culture. Despite some limitations, including the large number of items, time-consuming nature of the application, and the considerable number of items that could not be observed, our results suggest that the adapted version can be reliably used in Turkey. Evaluation of professionalism with such validated scales will ensure that the subject remains on the agenda of the parties in clinical training processes, and increase the quality of personal and institutional development and health services.

## Data Availability

The datasets used and/or analyzed during the current study are available from the corresponding author on reasonable request.

## Abbreviations

CFA: Confirmatory factor analysis
CFI: Comparative fit index
COSMIN: Consensus-based standards for the selection of health measurement instruments
Mini-Cex: Mini-clinical evaluation exercise
P-MEX: Professionalism Mini-evaluation Exercise
RMSEA: Root mean square error of approximation
TLI: Tucker-Lewis index
